# Supplementary material for: Development of a machine learning model for early prediction of plasma leakage in suspected dengue patients
Source: PLoS Negl Trop Dis. 2023 Mar 13;17(3):e0010758. doi: 10.1371/journal.pntd.0010758 (PMC10035900; doi:10.1371/journal.pntd.0010758)
Supplement: S1 Fig — The curves (decision curves) indicate the net benefit of the final model (DENV5F-AS) as well as three alternatives (classifying no patient as PL, classifying all patients as PL, and random guess) over the threshold of 0 to 1, and net benefit defined as (true positives)/N–(false positive/N)× the odds at the probability threshold (N: number of samples). Vertical blue dash line indicates the selected cut-off value of 0.5 for the predictions. (DOCX) [file pntd.0010758.s001.docx]

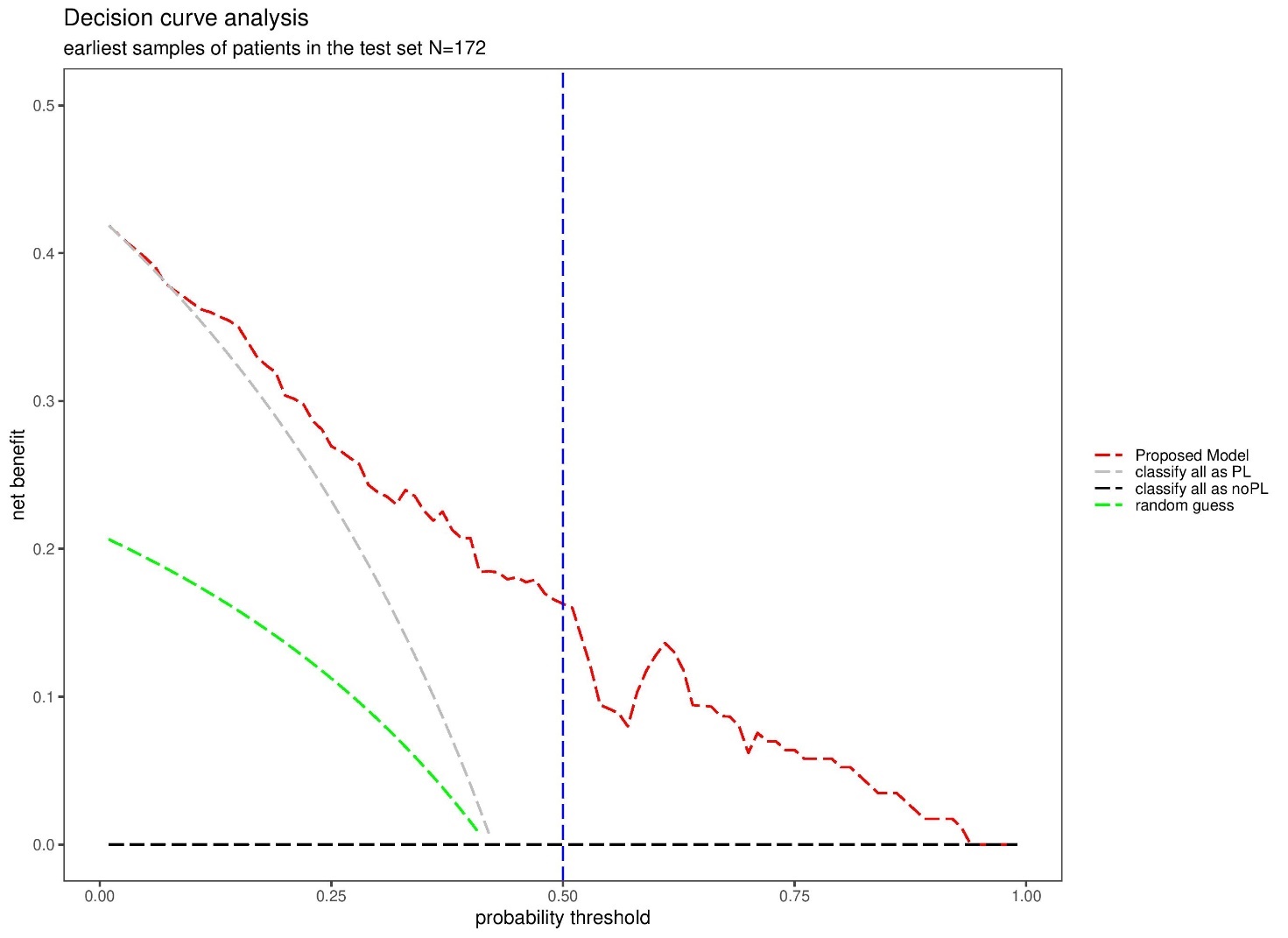


## S1 Fig - Decision curve analysis, where x-axis indicates the probability threshold to classify to PL (predicted probability≥ threshold) or noPL (otherwise), and y-axis indicates the net benefit limited to positive values. The curves (decision curves) indicate the net benefit of the final model (DENV_5F-AS_) as well as three alternatives (classifying no patient as PL, classifying all patients as PL, and random guess) over the threshold of 0 to 1, and net benefit defined as (true positives)/N – (false positive/N)× the odds at the probability threshold (N: number of samples). Vertical blue dash line indicates the selected cut-off value of 0.5 for the predictions.
