# Supplementary material for: Development of a machine learning model for early prediction of plasma leakage in suspected dengue patients
Source: PLoS Negl Trop Dis. 2023 Mar 13;17(3):e0010758. doi: 10.1371/journal.pntd.0010758 (PMC10035900; doi:10.1371/journal.pntd.0010758)
Supplement: S2 Fig — The features are sorted from top to bottom by their mean absolute SHAP values (higher interpreted as more contributing). Each point represents an instance and the connected lines across features belong to the same instance. For each feature the points are scattered perpendicular to the horizontal line to minimise overlapping. Feature values are normalised to [0 1] by the min-max normalisation method and colour-coded (grey points are missing values), outliers were squished to the range using Hampel filter. The colour of each line is the same as the value of the feature connected to in downwards direction. X-axis is the SHAP value computed for each instance. The order of the features is preserved from the original SHAP plot in Fig 4B) confusion matrix for each of the clusters for each matching cluster numbers (blue: all instances of the cluster, orange: earliest instance of patients in the cluster), the proportion of dengue and non-dengue patients are also captioned. (DOCX) [file pntd.0010758.s002.docx]

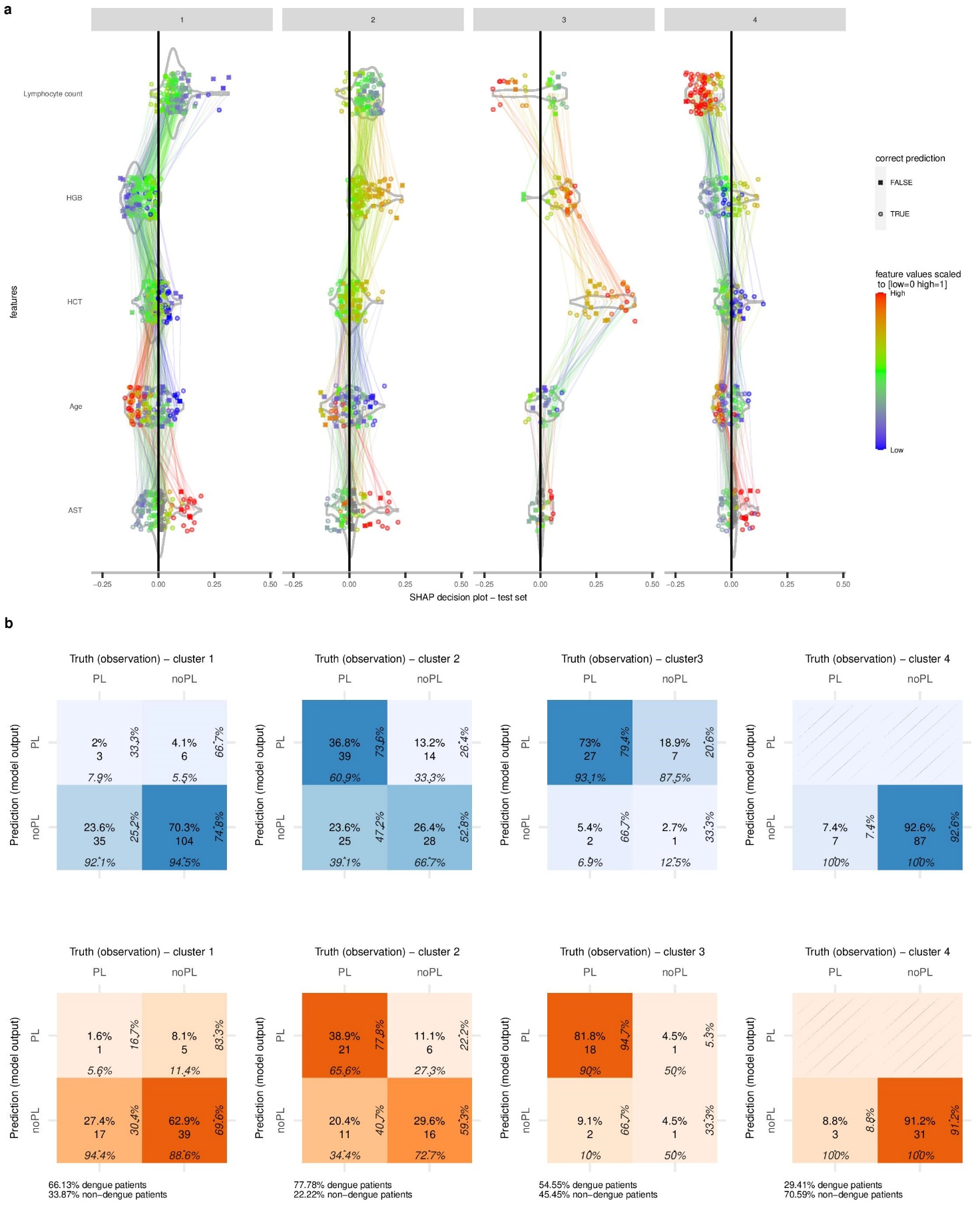


## S2 Fig model interpretability - a) SHAP decision plots for DENV_5F-AS_ on the test set in four clusters of SHAP values determined using the K-means method (k = 4). The features are sorted from top to bottom by their mean absolute SHAP values (higher interpreted as more contributing). Each point represents an instance and the connected lines across features belong to the same instance. For each feature the points are scattered perpendicular to the horizontal line to minimise overlapping. Feature values are normalised to [0 1] by the min-max normalisation method and colour-coded (grey points are missing values), outliers were squished to the range using Hampel filter. The colour of each line is the same as the value of the feature connected to in downwards direction. X-axis is the SHAP value computed for each instance. The order of the features is preserved from the original SHAP plot in Fig 4. b) confusion matrix for each of the clusters for each matching cluster numbers (blue: all instances of the cluster, orange: earliest instance of patients in the cluster), the proportion of dengue and non-dengue patients are also captioned.
