# Supplementary material for: Development of a machine learning model for early prediction of plasma leakage in suspected dengue patients
Source: PLoS Negl Trop Dis. 2023 Mar 13;17(3):e0010758. doi: 10.1371/journal.pntd.0010758 (PMC10035900; doi:10.1371/journal.pntd.0010758)
Supplement: S1 Table — (DOCX) [file pntd.0010758.s003.docx]

## S1 Table – Full feature list and description.

| **Source** | **Feature variable** | **Description** | **Data type / Unit of Measurement** |
| --- | --- | --- | --- |
| Basic Characteristics | study_id | Study ID number | ID number |
|  | dateandtimeofbloodcollectio | Date and time of blood collection | DD/MM/YYYY HH:MM |
|  | gender | Gender | Male / Female |
|  | age | Age | Years |
|  | district | District | City Name |
|  | heightcm | Height (cm) | Centimetre (cm) |
|  | weightkg | Weight (Kg) | Kilogram (Kg) |
|  | hospitaladmissiondateandtime | Hospital admission date and time | DD/MM/YYYY HH:MM |
|  | wardadmissiondateandtime | Ward admission date and time | DD/MM/YYYY HH:MM |
|  | dateandtimeofonsetoffever | Date and time of onset of fever | DD/MM/YYYY HH:MM |
|  | nsaidsgivenbeforehospitaladm | NSAIDS given before hospital admission | Yes / No |
|  | diabetes | Presence of Diabetes | Yes / No |
|  | hypertension | Presence of Hypertension | Yes / No |
|  | hyperlipidemia | Presence of Hyperlipidemia | Yes / No |
|  | cirrhosis | Presence of Cirrhosis | Yes / No |
|  | ckd | Presence of Chronic Kidney Disease | Yes / No |
|  | chf | Presence of Chronic heart failure | Yes / No |
|  | ihd | Presence of IHD | Yes / No |
|  | smoking | Smoking | Yes / No |
|  | pack_years | Pack years of smoking | No. of pack per year |
|  | alcohol_cons | Alcohol consumption | Yes / No |
|  | malignancy | Presence of Malignancy | Yes / No |
|  | allergy | Presence of Allergy | Yes / No |
|  | otherthanthisepisodehaveyou | Previous dengue episodes | Yes / No |
|  | dateandtimeofwarddischarge | Date and time of ward discharge | DD/MM/YYYY HH:MM |
|  | statusatwarddischarge | Status at ward discharge | Dead/Alive |
|  | plasma_leakage | Plasma leakage detected by ultrasound scanning (USS) and/or HCT rise | Yes / No |
|  | method_of_pl | Method of plasma leakage detection | USS / HCT rise / Both |
|  | pls_leakage_time | Date and time of plasma leakage | DD/MM/YYYY HH:MM |
|  | method_pl_us | Method of Plasma leakage detection by USS | Peritoneal fluid / Pleural Effusion / Both |
|  | gall_bladder | Gall bladder wall oedema detected by USS | Yes / No / Not Done |
|  | method_hct_rise | Method of plasma leakage detected by haematocrit rise | >20% rise / >45% overall / Both |
|  | compensatedshock | Compensated shock | Yes / No |
|  | uncompensatedshock | Uncompensated shock | Yes / No |
|  | severedengue | Severe dengue | Yes / No |
|  | dengue | Dengue/Non-Dengue | Dengue / Non-dengue |
|  | method_dengue_dia | Method of dengue diagnosis | RT-PCR / NS1 detection / Both |
|  | method_den_neg | Method of confirming negative for dengue | RT-PCR / NS1 detection / Both |
|  | serotype1 | Serotype of dengue virus (initial infection) | DENV Serotype (1,2,3 or 4) |
|  | quantitymeanpfuml1 | Viral load 1 | PFU/ml |
|  | serotype2 | Serotype of dengue virus (second infection, if any) | DENV Serotype (1,2,3,4 or NULL) |
|  | quantitymeanpfuml2 | Viral load 2 | PFU/ml |
|  | hosp_stay | Hospital stay duration | No. of days |
|  | complete | Completeness of data | Complete / Not complete |
| Dengue Signs and Symptoms | date_symptoms | Date of symptoms | DD/MM/YYYY |
|  | day_symptoms | Day of the symptoms measured from the onset of fever | No. of day post onset of fever |
|  | ab_pain | Abdominal pain | Yes / No |
|  | arthralgia | Arthralgia | Yes / No |
|  | bleeding | Bleeding | Yes / No |
|  | gum_bleed | Presence of gum bleeding | Yes / No |
|  | heamatomesis | Presence of heamatomesis | Yes / No |
|  | heamaturia | Presence of heamaturia | Yes / No |
|  | hemoptysis | Presence of hemoptysis | Yes / No |
|  | melena | Presence of melena | Yes / No |
|  | nose_bleed | Presence of nose bleeding | Yes / No |
|  | vaginal_bleed | Presence of vaginal bleeding | Yes / No |
|  | cough | Cough | Yes / No |
|  | chil_rigor | Chills or rigors | Yes / No |
|  | diarrhea | diarrhoea | Yes / No |
|  | dyspnea | Difficulty in breathing/dyspnea | Yes / No |
|  | headache | Headache | Yes / No |
|  | myalgia | Myalgia | Yes / No |
|  | nausea_vomit | Nausea or vomiting | Yes / No |
|  | retroorbital | Retro-orbital pain | Yes / No |
|  | hepatomegaly | Hepatomegaly | Yes / No |
|  | posturaldrop | Postural drop | Yes / No |
|  | Splenomegaly | Splenomegaly | Yes / No |
|  | Ankleedema | Ankle edema | Yes / No |
|  | symptom_score | Maximum symptom score out of the first three days of post onset of fever | Score (integer) |
| Full Blood Count | date_fbc | Date/Time of FBC report | DD/MM/YYYY |
|  | day_fbc | Day of the FBC from the onset of fever | No. of days post onset of fever |
|  | lowestwbc | Lowest White Blood cell count (WBC) each day | 10^3^ cells /µL |
|  | lowestneutrophil | Lowest Neutrophil absolute count each day | 10^3^ cells /µL |
|  | lowestlympho | Lowest Lymphocyte absolute count each day | 10^3^ cells /µL |
|  | lowesthgb | Lowest Hemoglobin count (HGB) each day | g/dL |
|  | highesthct | Highest Hematocrit count (HCT) each day | Percentage |
|  | lowestplatelets | Lowest Platelets count each day | 10^3^ cells /µL |
| Biochemical Metrics | date_biochemical | Date/Time of biochemical test report | DD/MM/YYYY HH:MM |
|  | day_biochemical | Day of the biochemical report from the onset of fever | No. of days post onset of fever |
|  | bloodsodium | Sodium level in blood | mmol/L |
|  | potassium | Potassium level in blood (K symbol for shorthand) | mmol/L |
|  | urea | Urea level in blood | mmol/L |
|  | creatinine | Creatinine level in blood | µmol/L |
|  | ast^a,b,c^ | Aspartate Aminotransferase (AST) level in blood | U/L |
|  | alt | Alanine Aminotransferase (ALT) level in blood | U/L |
|  | crp | C-reactive protein (CRP) level in blood | mg/L |
|  | cpk | Creatine phosphokinase (CPK) level in blood | U/L |
|  | totalbillirubin | Total bilirubin level in blood | µmol/L |
|  | directbilirubin | Direct bilirubin level in blood | µmol/L |
|  | alp | Alkaline phosphatase level (ALP) level in blood | U/L |
|  | gammagt | Gamma GT level in blood | U/L |
|  | amylase | Amylase level in blood | U/L |
|  | serumprotein | Serum protein level in blood | g/L |
|  | albumin | Albumin level in blood | g/L |
|  | globulin | Globulin level in blood | g/L |
|  | totalcholesterol | Total cholesterol level in blood | mg/dL |
|  | fastingplasmaglucose | Fasting plasma glucose level in blood | mg/dL |
|  | serumcalcium | Serum calcium level in blood | mmol/L |
|  | esr | Erythrocyte Sedimentation Rate (ESR) level in blood | mm/hour |
|  | troponin | Troponin level in blood | ng/mL |
| Ultrasound Data | date_us | Date and time of Ultra Sound (US) report | DD/MM/YYYY HH:MM |
|  | day_us | Day of the US from the onset of fever | No. of days post onset of fever |
|  | peritoneal_fluid | Peritoneal fluid | Yes / No |
|  | plu_effu | Plueral effusion | Yes / No |
|  | gall_bladder | Gall bladder wall oedema | Yes / No |
|  | us_negative | No change in USS report | Yes / No |
| Prothrombin Time | date_pt | Date and time of PT report | DD/MM/YYYY HH:MM |
|  | day_pt | Day of the PT from the onset of fever | No. of days post onset of fever |
|  | pts | Prothrombin Time (s) | Seconds |
|  | ptinr | Prothrombin Time (INR) | International normalised ratio (INR) |
|  | aptt | Activated partial thromboplastin time (APTT) | Seconds |
| **Note:** Observation Day not shown here due to feature being a merge of day_us, day_pt, day_symptom, day_fbc, day_biochemical | | | |
