# Supplementary material for: Development of a machine learning model for early prediction of plasma leakage in suspected dengue patients
Source: PLoS Negl Trop Dis. 2023 Mar 13;17(3):e0010758. doi: 10.1371/journal.pntd.0010758 (PMC10035900; doi:10.1371/journal.pntd.0010758)
Supplement: S2 Table — (DOCX) [file pntd.0010758.s004.docx]

## S2 Table - Filtered features (N = 43) for feature selection by Minimum Description Length (MDL) algorithm.

| **Source** | **Feature/variable** | **Data type / Unit of Measurement** |
| --- | --- | --- |
| Basic Characteristics | Observation Day^a^ | No. of days post onset of fever |
|  | Gender | Male / Female |
|  | **Age*** | Years |
|  | BMI^b^ | kg/m^2^ |
|  | DENV Serotype 2 | Yes/No |
|  | DENV Serotype 3 | Yes/No |
|  | DENV Serotype 4 | Yes/No |
|  | Hypertension | Yes/No |
| Dengue Signs and Symptoms | Arthralgia | Yes / No |
|  | Bleeding | Yes / No |
|  | Gum Bleeding | Yes / No |
|  | Haematemesis | Yes / No |
|  | Haematuria | Yes / No |
|  | Haemoptysis | Yes / No |
|  | Melena | Yes / No |
|  | Nosebleed | Yes / No |
|  | Vaginal bleed | Yes / No |
|  | Dyspnea | Yes / No |
|  | Retro-orbital pain | Yes / No |
|  | Hepatomegaly | Yes / No |
|  | Chills or rigors | Yes / No |
|  | Postural drop | Yes / No |
|  | Splenomegaly | Yes / No |
|  | Ankle edema | Yes / No |
|  | Headache | Yes / No |
|  | Cough | Yes / No |
|  | Diarrhea | Yes / No |
|  | Abdominal pain | Yes / No |
|  | Myalgia | Yes / No |
|  | Nausea or vomiting | Yes / No |
|  | Symptom score^c^ | Score (integer) |
| Full Blood Count | Leukocyte count | 10^3^ cells /µL |
|  | Neutrophil count | 10^3^ cells /µL |
|  | **Lymphocyte count^*^** | 10^3^ cells /µL |
|  | **Haemoglobin (HGB) count*** | g/dL |
|  | **Haematocrit (HCT) count *** | Percentage |
|  | Platelets count | 10^3^ cells /µL |
| Biochemical Metrics | Serum Sodium | mmol/L |
|  | Serum Potassium | mmol/L |
|  | Serum Creatinine | µmol/L |
|  | **Aspartate Aminotransferase (AST)*** | U/L |
|  | Serum C-reactive protein (CRP) | mg/L |
|  | Serum total bilirubin | µmol/L |
| (*****) - top five features selected by MDL | | |
| (a) - Merged feature of days post onset of fever in biochemical, ultrasound, prothrombin time, FBC and Symptoms data set | | |
| (b) – Body Mass Index | | |
| (c) - Maximum symptoms score out of the first four days of post onset of fever. The score is calculated as the number of existing symptoms. | | |
