# Supplementary material for: Development of a machine learning model for early prediction of plasma leakage in suspected dengue patients
Source: PLoS Negl Trop Dis. 2023 Mar 13;17(3):e0010758. doi: 10.1371/journal.pntd.0010758 (PMC10035900; doi:10.1371/journal.pntd.0010758)
Supplement: S3 Table — (DOCX) [file pntd.0010758.s005.docx]

## S3 Table - Performance metrics on the test set using different number of selected features using Minimum Description Length (MDL) algorithm.

| **number of selected features** | **MCC** | **BA** | **PPV** | **NPV** | **sensitivity** | **specificity** | **AUC** | **PRAUC** | **Brier** |
| --- | --- | --- | --- | --- | --- | --- | --- | --- | --- |
| 4 | 0.34 | 0.64 | 0.7 | 0.72 | 0.36 | 0.91 | 0.77 | 0.68 | 0.18 |
| 5^a^ | 0.43 | 0.7 | 0.72 | 0.76 | 0.5 | 0.89 | 0.8 | 0.69 | 0.17 |
| 6 | 0.41 | 0.68 | 0.73 | 0.74 | 0.45 | 0.9 | 0.8 | 0.7 | 0.17 |
| 7 | 0.4 | 0.68 | 0.69 | 0.75 | 0.49 | 0.88 | 0.79 | 0.7 | 0.17 |
| 8 | 0.4 | 0.68 | 0.72 | 0.74 | 0.46 | 0.9 | 0.81 | 0.73 | 0.17 |
| 9 | 0.38 | 0.66 | 0.73 | 0.73 | 0.4 | 0.92 | 0.8 | 0.71 | 0.17 |
| 10 | 0.4 | 0.67 | 0.72 | 0.74 | 0.45 | 0.9 | 0.79 | 0.7 | 0.18 |
| 21^b^ | 0.39 | 0.67 | 0.72 | 0.74 | 0.43 | 0.91 | 0.78 | 0.69 | 0.18 |
| **average** | **0.39** | **0.67** | **0.72** | **0.74** | **0.44** | **0.90** | **0.79** | **0.70** | **0.17** |

AUC: area under the receiver operating characteristics curve, PRAUC: area under the precision-recall curve, balanced accuracy BA = ((TP/(TP+FN)+TN/(TN+FP)))⁄2, negative predictive value (NPV) = TN/(TN+FN) , positive predictive value (PPV) = TP/(TP+FP) , Recall = TP/(TP+FN), sensitivity = TP/(TP+FN), specificity = TN/(TN+FP), Matthews correlation coefficient (MCC) = (TP×TN-FP×FN)/√((TP+FP)(TP+FN)(TN+FP)(TN+FN)), Brier = mean squared error between predicted probabilities and the observed values (positive plasma leakage = 1, negative plasma leakage = 0). TP, TN, FP, and FN are true positives, true negatives, false positives, and false negatives, respectively.
(a) number of selected features chosen for the proposed model
(b) all positively scored features
