# Supplementary material for: Development of a machine learning model for early prediction of plasma leakage in suspected dengue patients
Source: PLoS Negl Trop Dis. 2023 Mar 13;17(3):e0010758. doi: 10.1371/journal.pntd.0010758 (PMC10035900; doi:10.1371/journal.pntd.0010758)
Supplement: S4 Table — (DOCX) [file pntd.0010758.s006.docx]

## S4 Table - Performance metrics on the test set using 10 different seeds based on "L'Ecuyer-CMRG" seeding in R version 4.1.2.

| **Seed** | **MCC** | **BA** | **PPV** | **NPV** | **sensitivity** | **specificity** | **AUC** | **PRAUC** | **Brier** |
| --- | --- | --- | --- | --- | --- | --- | --- | --- | --- |
| 69 | 0.30 | 0.64 | 0.54 | 0.79 | 0.43 | 0.85 | 0.71 | 0.55 | 0.18 |
| 246* | 0.43 | 0.70 | 0.72 | 0.76 | 0.50 | 0.89 | 0.80 | 0.69 | 0.17 |
| 456 | 0.38 | 0.68 | 0.61 | 0.78 | 0.53 | 0.83 | 0.78 | 0.61 | 0.18 |
| 789 | 0.31 | 0.64 | 0.62 | 0.72 | 0.42 | 0.86 | 0.74 | 0.61 | 0.19 |
| 3197 | 0.34 | 0.65 | 0.65 | 0.74 | 0.43 | 0.87 | 0.74 | 0.65 | 0.19 |
| 3659 | 0.40 | 0.68 | 0.62 | 0.81 | 0.49 | 0.88 | 0.78 | 0.63 | 0.16 |
| 4065 | 0.24 | 0.60 | 0.57 | 0.72 | 0.33 | 0.87 | 0.69 | 0.56 | 0.20 |
| 7193 | 0.37 | 0.66 | 0.71 | 0.72 | 0.42 | 0.90 | 0.79 | 0.71 | 0.18 |
| 10736 | 0.32 | 0.62 | 0.73 | 0.70 | 0.30 | 0.94 | 0.68 | 0.62 | 0.21 |
| 445 | 0.24 | 0.60 | 0.61 | 0.69 | 0.31 | 0.89 | 0.67 | 0.57 | 0.21 |
| **average** | **0.43** | **0.69** | **0.63** | **0.86** | **0.46** | **0.93** | **0.81** | **0.59** | **0.13** |

AUC: area under the receiver operating characteristics curve, PRAUC: area under the precision-recall curve, balanced accuracy BA = ((TP/(TP+FN)+TN/(TN+FP)))⁄2, negative predictive value (NPV) = TN/(TN+FN) , positive predictive value (PPV) = TP/(TP+FP) , Recall = TP/(TP+FN), sensitivity = TP/(TP+FN), specificity = TN/(TN+FP), Matthews correlation coefficient (MCC) = (TP×TN-FP×FN)/√((TP+FP)(TP+FN)(TN+FP)(TN+FN)), Brier = mean squared error between predicted probabilities and the observed values (positive plasma leakage = 1, negative plasma leakage = 0). TP, TN, FP, and FN are true positives, true negatives, false positives, and false negatives, respectively.
(*) The seed used for the proposed model
