# Supplementary material for: Development of a machine learning model for early prediction of plasma leakage in suspected dengue patients
Source: PLoS Negl Trop Dis. 2023 Mar 13;17(3):e0010758. doi: 10.1371/journal.pntd.0010758 (PMC10035900; doi:10.1371/journal.pntd.0010758)
Supplement: S5 Table — (DOCX) [file pntd.0010758.s007.docx]

## S5 Table - Selected features using 10 different seeds.

| **Seed** | **AST** | **HCT** | **HGB** | **lowestneutrophil** | **lowestwbc** | **Age** | **Gender** | **Lymphocyte count** | **CRP** |
| --- | --- | --- | --- | --- | --- | --- | --- | --- | --- |
| 69 | 1 | 1 | 1 | 1 | 1 | 0 | 0 | 0 | 0 |
| 246* | 1 | 1 | 1 | 0 | 0 | 1 | 0 | 1 | 0 |
| 445 | 1 | 1 | 1 | 0 | 0 | 0 | 1 | 0 | 1 |
| 456 | 0 | 1 | 1 | 0 | 0 | 1 | 1 | 1 | 0 |
| 789 | 1 | 1 | 1 | 0 | 1 | 0 | 0 | 1 | 0 |
| 3197 | 1 | 1 | 1 | 0 | 1 | 1 | 0 | 0 | 0 |
| 3659 | 1 | 1 | 1 | 0 | 0 | 0 | 1 | 1 | 0 |
| 4065 | 1 | 1 | 1 | 0 | 0 | 1 | 0 | 0 | 1 |
| 7193 | 0 | 1 | 1 | 0 | 0 | 1 | 0 | 1 | 1 |
| 10736 | 1 | 1 | 1 | 0 | 0 | 0 | 1 | 0 | 1 |
| **Average** | **0.8** | **1** | **1** | **0.1** | **0.3** | **0.5** | **0.4** | **0.5** | **0.4** |
| **Note**: Selected features are indicated by 1 and features not selected are indicated by 0. Feature selection was done by Minimum Description Length (MDL) algorithm. (*) The seed used for the proposed model | | | | | | | | | |
