# Supplementary material for: Development of a machine learning model for early prediction of plasma leakage in suspected dengue patients
Source: PLoS Negl Trop Dis. 2023 Mar 13;17(3):e0010758. doi: 10.1371/journal.pntd.0010758 (PMC10035900; doi:10.1371/journal.pntd.0010758)
Supplement: S6 Table — (DOCX) [file pntd.0010758.s008.docx]

## S6 Table - Summary statistics of feature values in each cluster.

|  | **HCT^a^** | **HGB ^b^** | **AST ^c^** | **Lymphocyte count ^d^** | **Age ^e^** |
| --- | --- | --- | --- | --- | --- |
| **Cluster 1** |  |  |  |  |  |
| 1st Quartile | 34 | 11.1 | 28 | 0.46 | 22 |
| Median | 39 | 12.0 | 37 | 0.67 | 32 |
| 3rd Quartile | 41 | 12.5 | 55 | 0.86 | 49 |
| **Cluster 2** |  |  |  |  |  |
| 1st Quartile | 41 | 13.4 | 33 | 0.50 | 21 |
| Median | 42 | 13.9 | 43 | 0.67 | 25 |
| 3rd Quartile | 43 | 14.3 | 69 | 0.87 | 36 |
| **Cluster 3** |  |  |  |  |  |
| 1st Quartile | 44 | 14.0 | 31 | 0.69 | 23 |
| Median | 45 | 14.6 | 47 | 0.98 | 31 |
| 3rd Quartile | 47 | 15.4 | 73 | 1.48 | 35 |
| **Cluster 4** |  |  |  |  |  |
| 1st Quartile | 33 | 10.9 | 31 | 1.35 | 21 |
| Median | 37 | 11.9 | 42 | 1.74 | 30 |
| 3rd Quartile | 40 | 13.2 | 103 | 2.36 | 49 |
| (a) HCT in percentage  (b) HGB in g/DL  (c) AST in U/L  (d) Lowest Lymphocyte absolute count each day 10^3^ cells /µL  (e) patient age in years | | | | | |
|  | | | | | |
