# Supplementary material for: Development of a machine learning model for early prediction of plasma leakage in suspected dengue patients
Source: PLoS Negl Trop Dis. 2023 Mar 13;17(3):e0010758. doi: 10.1371/journal.pntd.0010758 (PMC10035900; doi:10.1371/journal.pntd.0010758)
Supplement: S7 Table — The imputation resulted in the selection of HCT, HGB. Gender, age, and AST as selected features. (DOCX) [file pntd.0010758.s009.docx]

## S7 Table - Performance metrics on the test set for a final model developed by the development set with imputed missingness using conditional multiple imputation from *mice* package. The seeding based on "L'Ecuyer-CMRG" seeding in R version 4.1.2. The imputation resulted in the selection of HCT, HGB. Gender, age, and AST as selected features.

| **Seed** | **MCC** | **BA** | **PPV** | **NPV** | **sensitivity** | **specificity** | **AUC** | **PRAUC** | **Brier** |
| --- | --- | --- | --- | --- | --- | --- | --- | --- | --- |
| 246* | 0.21 | 0.60 | 0.52 | 0.70 | 0.43 | 0.77 | 0.69 | 0.55 | 0.22 |

*same seeding used to make it comparable to the proposed implementation in which the missingness was handled by the learners.
