# Supplementary material for: Development of a machine learning model for early prediction of plasma leakage in suspected dengue patients
Source: PLoS Negl Trop Dis. 2023 Mar 13;17(3):e0010758. doi: 10.1371/journal.pntd.0010758 (PMC10035900; doi:10.1371/journal.pntd.0010758)
Supplement: S8 Table — (DOCX) [file pntd.0010758.s010.docx]

## S8 Table - R package list.

| **Package** | **Version** | **Reference** |
| --- | --- | --- |
| arsenal | 3.6.3 | Ethan Heinzen, Jason Sinnwell, Elizabeth Atkinson, Tina Gunderson and Gregory Dougherty (2021). arsenal: An Arsenal of 'R' Functions for Large-Scale Statistical Summaries. R package version 3.6.3. https://CRAN.R-project.org/package=arsenal |
| bestNormalize | 1.8.3 | (1) Peterson, R. A. (2021). Finding Optimal Normalizing Transformations via bestNormalize. The R Journal, 13:1, 310-329, DOI:10.32614/RJ-2021-041 |
| broom | 1.0.0 | David Robinson, Alex Hayes and Simon Couch (2022). broom: Convert Statistical Objects into Tidy Tibbles. R package version 1.0.0. https://CRAN.R-project.org/package=broom |
| cluster | 2.1.2 | Maechler, M., Rousseeuw, P., Struyf, A., Hubert, M., Hornik, K.(2021). cluster: Cluster Analysis Basics and Extensions. R package version 2.1.2. |
| CORElearn | 1.56.0 | Marko Robnik-Sikonja and Petr Savicky (2021). CORElearn: Classification, Regression and Feature Evaluation. R package version 1.56.0. https://CRAN.R-project.org/package=CORElearn |
| cowplot | 1.1.1 | Claus O. Wilke (2020). cowplot: Streamlined Plot Theme and Plot Annotations for 'ggplot2'. R package version 1.1.1. https://CRAN.R-project.org/package=cowplot |
| cvms | 1.3.4 | Ludvig Renbo Olsen and Hugh Benjamin Zachariae (2022). cvms: Cross-Validation for Model Selection. R package version 1.3.4. https://CRAN.R-project.org/package=cvms |
| data.table | 1.14.2 | Matt Dowle and Arun Srinivasan (2021). data.table: Extension of `data.frame`. R package version 1.14.2. https://CRAN.R-project.org/package=data.table |
| dendextend | 1.16.0 | Tal Galili (2015). dendextend: an R package for visualizing, adjusting, and comparing trees of hierarchical clustering. Bioinformatics. DOI: 10.1093/bioinformatics/btv428 |
| DescTools | 0.99.45 | Andri Signorell et mult. al. (2022). DescTools: Tools for descriptive statistics. R package version 0.99.45. |
| dplyr | 1.0.9 | Hadley Wickham, Romain François, Lionel Henry and Kirill Müller (2022). dplyr: A Grammar of Data Manipulation. R package version 1.0.9. https://CRAN.R-project.org/package=dplyr |
| egg | 0.4.5 | Baptiste Auguie (2019). egg: Extensions for 'ggplot2': Custom Geom, Custom Themes, Plot Alignment, Labelled Panels, Symmetric Scales, and Fixed Panel Size. R package version 0.4.5. https://CRAN.R-project.org/package=egg |
| factoextra | 1.0.7 | Alboukadel Kassambara and Fabian Mundt (2020). factoextra: Extract and Visualize the Results of Multivariate Data Analyses. R package version 1.0.7. https://CRAN.R-project.org/package=factoextra |
| fairness | 1.2.2 | Nikita Kozodoi and Tibor V. Varga (2021). fairness: Algorithmic Fairness Metrics. R package version 1.2.2. https://CRAN.R-project.org/package=fairness |
| forcats | 0.5.1 | Hadley Wickham (2021). forcats: Tools for Working with Categorical Variables (Factors). R package version 0.5.1. https://CRAN.R-project.org/package=forcats |
| FSelectorRcpp | 0.3.8 | Zygmunt Zawadzki and Marcin Kosinski (2021). FSelectorRcpp: 'Rcpp' Implementation of 'FSelector' Entropy-Based Feature Selection Algorithms with a Sparse Matrix Support. R package version 0.3.8. https://CRAN.R-project.org/package=FSelectorRcpp |
| ggforce | 0.3.3 | Thomas Lin Pedersen (2021). ggforce: Accelerating 'ggplot2'. R package version 0.3.3. https://CRAN.R-project.org/package=ggforce |
| ggimage | 0.3.1 | Guangchuang Yu (2022). ggimage: Use Image in 'ggplot2'. R package version 0.3.1. https://CRAN.R-project.org/package=ggimage |
| ggplot2 | 3.3.6 | H. Wickham. ggplot2: Elegant Graphics for Data Analysis. Springer-Verlag New York, 2016. |
| ggpmisc | 0.4.7 | Pedro J. Aphalo (2022). ggpmisc: Miscellaneous Extensions to 'ggplot2'. R package version 0.4.7. https://CRAN.R-project.org/package=ggpmisc |
| ggpp | 0.4.4 | Pedro J. Aphalo (2022). ggpp: Grammar Extensions to 'ggplot2'. R package version 0.4.4. https://CRAN.R-project.org/package=ggpp |
| ggpubr | 0.4.0 | Alboukadel Kassambara (2020). ggpubr: 'ggplot2' Based Publication Ready Plots. R package version 0.4.0. https://CRAN.R-project.org/package=ggpubr |
| ggROC | 1.0 | Honglong Wu (2013). ggROC: package for roc curve plot with ggplot2. R package version 1.0. https://CRAN.R-project.org/package=ggROC |
| gridExtra | 2.3 | Baptiste Auguie (2017). gridExtra: Miscellaneous Functions for "Grid" Graphics. R package version 2.3. https://CRAN.R-project.org/package=gridExtra |
| iml | 0.11.0 | Molnar C, Bischl B, Casalicchio G (2018). “iml: An R package for Interpretable Machine Learning.” _JOSS_, *3*(26), 786. doi: 10.21105/joss.00786 (URL: https://doi.org/10.21105/joss.00786),<URL: https://joss.theoj.org/papers/10.21105/joss.00786>. |
| MLmetrics | 1.1.1 | Yachen Yan (2016). MLmetrics: Machine Learning Evaluation Metrics. R package version 1.1.1. https://CRAN.R-project.org/package=MLmetrics |
| mlr3 | 0.14.0 | Lang M, Binder M, Richter J, Schratz P, Pfisterer F, Coors S, Au Q, Casalicchio G, Kotthoff L, Bischl B (2019). “mlr3: A modern object-oriented machine learning framework in R.” _Journal ofOpen Source Software_. doi: 10.21105/joss.01903 (URL: https://doi.org/10.21105/joss.01903), <URL: https://joss.theoj.org/papers/10.21105/joss.01903>. |
| mlr3extralearners | 0.5.18 | Raphael Sonabend and Patrick Schratz (2022). mlr3extralearners: Extra Learners For mlr3. R package version 0.5.18. |
| mlr3filters | 0.5.0 | Patrick Schratz, Michel Lang, Bernd Bischl and Martin Binder (2022). mlr3filters: Filter Based Feature Selection for 'mlr3'. R package version 0.5.0. https://CRAN.R-project.org/package=mlr3filters |
| mlr3learners | 0.5.3 | Michel Lang, Quay Au, Stefan Coors and Patrick Schratz (2022). mlr3learners: Recommended Learners for 'mlr3'. R package version 0.5.3. https://CRAN.R-project.org/package=mlr3learners |
| mlr3pipelines | 0.4.1 | Binder M, Pfisterer F, Lang M, Schneider L, Kotthoff L, Bischl B (2021). “mlr3pipelines - Flexible Machine Learning Pipelines in R.” _Journal of Machine Learning Research_, *22*(184), 1-7.<URL: https://jmlr.org/papers/v22/21-0281.html>. |
| mlr3tuning | 0.13.1 | Marc Becker, Michel Lang, Jakob Richter, Bernd Bischl and Daniel Schalk (2022). mlr3tuning: Tuning for 'mlr3'. R package version 0.13.1. https://CRAN.R-project.org/package=mlr3tuning |
| mltools | 0.3.5 | Ben Gorman (2018). mltools: Machine Learning Tools. R package version 0.3.5. https://CRAN.R-project.org/package=mltools |
| mice | 3.14.0 | van Buuren S, Groothuis-Oudshoorn K (2011). “mice: Multivariate Imputation by Chained Equations in R.” *Journal of Statistical Software*, **45**(3), 1-67. [doi:10.18637/jss.v045.i03](https://doi.org/10.18637/jss.v045.i03) |
| paradox | 0.10.0 | Michel Lang, Bernd Bischl, Jakob Richter, Xudong Sun and Martin Binder (2022). paradox: Define and Work with Parameter Spaces for Complex Algorithms. R package version 0.10.0. https://CRAN.R-project.org/package=paradox |
| parallelMap | 1.5.1 | Bernd Bischl, Michel Lang and Patrick Schratz (2021). parallelMap: Unified Interface to Parallelization Back-Ends. R package version 1.5.1. https://CRAN.R-project.org/package=parallelMap |
| Plumber | 1.2.1 | Schloerke B, Allen J (2022). *plumber: An API Generator for R*. https://www.rplumber.io, https://github.com/rstudio/plumber |
| praznik | 11.0.0 | Miron B. Kursa (2021). Praznik: High performance information-based feature selection. SoftwareX, 16, 100819. URL https://doi.org/10.1016/j.softx.2021.100819 |
| pROC | 1.18.0 | Xavier Robin, Natacha Turck, Alexandre Hainard, Natalia Tiberti, Frédérique Lisacek, Jean-Charles Sanchez and Markus Müller (2011). pROC: an open-source package for R and S+ to analyze and compare ROC curves. BMC Bioinformatics, 12, p. 77. DOI: 10.1186/1471-2105-12-77 <http://www.biomedcentral.com/1471-2105/12/77/> |
| purrr | 0.3.4 | Lionel Henry and Hadley Wickham (2020). purrr: Functional Programming Tools. R package version 0.3.4. https://CRAN.R-project.org/package=purrr |
| R | 4.1.2 | R Core Team (2021). R: A language and environment for statistical computing. R Foundation for Statistical Computing, Vienna, Austria. URL https://www.R-project.org/. |
| ranger | 0.14.1 | Marvin N. Wright, Andreas Ziegler (2017). ranger: A Fast Implementation of Random Forests for High Dimensional Data in C++ and R. Journal of Statistical Software, 77(1), 1-17. doi:10.18637/jss.v077.i01 |
| readr | 2.1.2 | Hadley Wickham, Jim Hester and Jennifer Bryan (2022). readr: Read Rectangular Text Data. R package version 2.1.2. https://CRAN.R-project.org/package=readr |
| readxl | 1.4.0 | Hadley Wickham and Jennifer Bryan (2022). readxl: Read Excel Files. R package version 1.4.0. https://CRAN.R-project.org/package=readxl |
| report | 0.5.1 | Makowski, D., Ben-Shachar, M.S., Patil, I. & Lüdecke, D. (2020). Automated Results Reporting as a Practical Tool to Improve Reproducibility and Methodological Best Practices Adoption. CRAN. Available from https://github.com/easystats/report. doi: . |
| repr | 1.1.4 | Philipp Angerer, Thomas Kluyver and Jan Schulz (2022). repr: Serializable Representations. R package version 1.1.4. https://CRAN.R-project.org/package=repr |
| rsvg | 2.3.1 | Jeroen Ooms (2022). rsvg: Render SVG Images into PDF, PNG, (Encapsulated) PostScript, or Bitmap Arrays. R package version 2.3.1. https://CRAN.R-project.org/package=rsvg |
| stringr | 1.4.0 | Hadley Wickham (2019). stringr: Simple, Consistent Wrappers for Common String Operations. R package version 1.4.0. https://CRAN.R-project.org/package=stringr |
| tibble | 3.1.8 | Kirill Müller and Hadley Wickham (2022). tibble: Simple Data Frames. R package version 3.1.8. https://CRAN.R-project.org/package=tibble |
| tidyr | 1.2.0 | Hadley Wickham and Maximilian Girlich (2022). tidyr: Tidy Messy Data. R package version 1.2.0. https://CRAN.R-project.org/package=tidyr |
| tidyverse | 1.3.2 | Wickham H, Averick M, Bryan J, Chang W, McGowan LD, François R, Grolemund G, Hayes A, Henry L, Hester J, Kuhn M, Pedersen TL, Miller E, Bache SM, Müller K, Ooms J, Robinson D, Seidel DP,Spinu V, Takahashi K, Vaughan D, Wilke C, Woo K, Yutani H (2019). “Welcome to the tidyverse.” _Journal of Open Source Software_, *4*(43), 1686. doi: 10.21105/joss.01686 (URL:https://doi.org/10.21105/joss.01686). |
| tsutils | 0.9.3 | Nikolaos Kourentzes (2022). tsutils: Time Series Exploration, Modelling and Forecasting. R package version 0.9.3. https://CRAN.R-project.org/package=tsutils |
| writexl | 1.4.0 | Jeroen Ooms (2021). writexl: Export Data Frames to Excel 'xlsx' Format. R package version 1.4.0. https://CRAN.R-project.org/package=writexl |
