# Supplementary material for: Development of a machine learning model for early prediction of plasma leakage in suspected dengue patients
Source: PLoS Negl Trop Dis. 2023 Mar 13;17(3):e0010758. doi: 10.1371/journal.pntd.0010758 (PMC10035900; doi:10.1371/journal.pntd.0010758)
Supplement: S1 Text — (DOCX) [file pntd.0010758.s011.docx]

## Selected hyperparameters in the final model DENV_5F-AS_

**Random forest:** rf_ss.frac=1, rf_ss.stratify=FALSE, rf_ss.replace=FALSE, rf.ntree=65, rf.mtry=1, rf.nodesize=2, rf.na.action=na.impute, rf.seed=-246.

**LightGBM:** lgbm_ss.frac=1, lgbm_ss.stratify=FALSE, lgbm_ss.replace=FALSE, lgbm.verbose=-1, lgbm.num_threads=1, lgbm.min_data_in_leaf=12, lgbm.min_sum_hessian_in_leaf=0.041, lgbm.bagging_freq=0, lgbm.lambda_l1=0.7179, lgbm.lambda_l2=0.0508, lgbm.min_gain_to_split=0.272, lgbm.min_data_per_group=28, lgbm.max_bin=244, lgbm.min_data_in_bin=8.

## Handling of missingness by learners

For the random forest model, the values for a variable with missingness are randomly drawn from non-missing in-bag data when a node splits on a variable with missing data. For LightGBM, missing values for continuous variables are assigned to the side of the splits that most reduces the loss.

## Description of performance metrics used to evaluate our models

The performance of our model was calculated in terms of Matthews correlation coefficient (MCC). The cut-off value for the probability threshold as model outputs was chosen to be 0.5, where values greater than the threshold are classified as PL (positive class) and noPL (negative class) otherwise. In addition, confusion matrix, balanced accuracy (BA), area under the receiver operating characteristics curve (AUC), positive predictive value (PPV), negative predictive value (NPV), sensitivity, specificity, area under precision-recall curve (PRAUC), and Brier score are additional measurements applied to construct a multi-metric assessment for model performance. For Brier score, observed values were 1 for plasma leakage and 0 for not plasma leakage. TP, TN, FP, and FN denote True Positive, True Negative, False Positive, and False Negative, respectively. The metrics are described as:

$$MCC= \frac{TP\times TN-FP\times FN}{\sqrt{\left( TP+FP \right)\times\left( TP+FN \right)\times\left( TN+FP \right)\times\left( TN+FN \right)}}$$

$$BA= \frac{\frac{TP}{\left( TP+FN \right)}+\frac{TN}{\left( TN+FP \right)}}{2}$$

$$PPV=\frac{TP}{(TP+FP)}$$

$$NPV= \frac{TN}{(TN+FN)}$$

$$Sensitivity= \frac{TP}{(TP+FN)}$$

$$Specificity= \frac{TN}{(TN+FP)}$$

$$Brier Score= \frac{1}{N}\sum_{t=1}^{N} \left( {predicted probability}_{t}-{observed values}_{t} \right)^{2}$$

The theoretical chance level (random guess based on no prior knowledge) in binary classification is defined as MCC = 0 and BA = 0.5.

## Model consistency

As a post-prediction step to evaluate the final model, abstention windows were applied to the predictions on the test set to assess the prediction performance by enforcing restrictions on acceptable ranges of predicted probabilities to be assigned to classes. Abstention window size varied from 0 to 1. Two hundred windows were randomly generated and applied symmetrically around the decision threshold of 0.5. The instances prediction probabilities falling within the abstention window were counted as unclassified, i.e.

$Predicted Class= \left\{ \begin{aligned} PL if PP>\left( 0.5+\frac{AW}{2} \right) \\ unclassified if\left( 0.5-\frac{AW}{2} \right)<PP\leq\left( 0.5+\frac{AW}{2} \right) \\ noPL if PP\leq\left( 0.5-\frac{AW}{2} \right) \end{aligned} \right.$

where $PP$ and $AW$ refer to predicted probabilities (model output) and abstention windows, respectively. To evaluate performance metrics in presence of abstention windows, they were multiplied by the ratio of classified instances to total number of instances resulting in weighted performance metrics (e.g., weighted MCC). Applying the abstention windows did not lead to increasing performance as no positive correlation between the weighted performance metrics and the abstention windows was found.

There was consistency in model development using 10 different randomised selection of patients for data split (S4 Table). None of the performance metrics was found to contain an outlier (Grubbs’s test). In addition, the seeding demonstrated consistency in feature selection. HCT and HGB were selected 100% of the times, AST was selected in 80% seeds, while age and lymphocyte count were selected in 50% of the experiments by seeds (S5 Table).

## Clustering of SHAP values

We applied K-means clustering algorithm based on the Hartigan-Wong algorithm to extract four subgroups of patients contributing differently to the prediction of plasma leakage. Within-cluster sum of squares (SS) of clusters 1-4 are 2.00, 1.41, 0.83, 0.90 respectively. The ratio of SS between-clusters to SS within-clusters is 60.5% calculated as *(between SS)/(total SS)*. Fig S2 illustrates the splitting of patients to four clusters to better visualize distinct patterns in feature contributions and interactions between features. The distribution of feature values within each cluster is outlined in S6 Table. A new patient record (instance) can therefore be assigned to one of the clusters using a relevant distance metric (e.g., Euclidean) to the centre of clusters. Confusion matrices for the samples in each cluster are also shown to have an estimate of expected performance for a new patient record assigned to a cluster.
