# Supplementary material for: Development of a machine learning model for early prediction of plasma leakage in suspected dengue patients
Source: PLoS Negl Trop Dis. 2023 Mar 13;17(3):e0010758. doi: 10.1371/journal.pntd.0010758 (PMC10035900; doi:10.1371/journal.pntd.0010758)
Supplement: Attachment — Submitted filename: PNTD-D-22-01084-reviewer-comments.pdf [file pntd.0010758.s012.pdf]

Reviewer comments for PNTD-D-22-01084

Paper title "Development of a machine learning model for early prediction of plasma leakage in suspected dengue patients"

Paper authors Zargari Marandi et al.

Summary The paper develops a machine learning model to predict plasma leakage in suspected dengue patients using data from a prospective cohort study in Sri Lanka. It is clinically important to identify predictors that detect plasma leakage in the first few days dengue infection to improve triage. A rigorous decision curve analysis is performed on the overall sample and by dengue diagnosis subgroup, and the model is interpreted using Shapley additive explanations.

The authors may consider new analyses for (1) a multi-label classification task (i.e., PL, noPL, not recorded); and (2) retaining variables with missing values (that are not completely missing in the training set), or imputing these values prior to classification. These suggestions are explained below.

Major comments

1. Why was plasma leakage not recorded in 172 patients? As reported in Table 1, there is a much higher proportion of missing outcome values in the non-dengue patients (26%) compared to the dengue patients (16%), which suggests the missing values are not missing at random and may indicate information of clinical importance. In this case, it might be more realistic to treat the prediction problem as a multi-class problem (i.e., PL, noPL, not recorded).
2. I'm unsure of the claim that removing instances with more than 50% of the features missing would reduce biased interpretation of feature contributions. Removing these instances would almost surely bias the interpretation because the missing values are presumably not missing at random. There are generally higher proportions of missing feature values among the non-dengue patients compared to the dengue patients (Table 1), presumably because the tests are given more to the sicker patients. I expect that excluding these instances, rather than let the algorithms handle the missing data internally or impute the values, would limit the ability of the classifiers to learn the task. Several papers show that imputing the missing values (e.g., with k-NN) can outperform the internal methods used by decision tree-based algorithms to treat missing data (e.g., `https://www.tandfonline.com/doi/pdf/10.1080/08839514.2018.1448143`). This paper shows that adding missing-data perturbation prior to imputation can actually improve prediction accuracy in supervised classification tasks by regularizing the classifier.

3. Relatedly, in the discussion two contradictory statements are made: that the proposed classifier can handle missing data and later, that many variables had be excluded due to missingness. I can see why variables that are completely missing in the training set (Dengue virus serotype and viral load) need to be dropped, but not understand the intuition for dropping any other variables.

Minor comments

1. Pg. 4, typo: "focusses"
2. Pg. 5: I don't understand why the viral load and ultrasonography predictors are removed due to high costs. Is this because the non-dengue patients don't get tested? Why are the ultrasound predictors not summarized in Table 1?
3. Table 2: how is the variance of the performance metrics calculated?
4. Figure 3a.: Should the x-axis be labeled instead "1-Specificity" (the FPR)?
