# Supplementary material for: Development of a machine learning model for early prediction of plasma leakage in suspected dengue patients
Source: PLoS Negl Trop Dis. 2023 Mar 13;17(3):e0010758. doi: 10.1371/journal.pntd.0010758 (PMC10035900; doi:10.1371/journal.pntd.0010758)
Supplement: Attachment — Submitted filename: Response_to_reviewers.docx [file pntd.0010758.s013.docx]

Reviewer #1: Summary
The paper develops a machine learning model to predict plasma leakage in suspected dengue patients using data from a prospective cohort study in Sri Lanka. It is clinically important to identify predictors that detect plasma leakage in the first few days dengue infection to improve triage. A rigorous decision curve analysis is performed on the overall sample and by dengue diagnosis subgroup, and the model is interpreted using
Shapley additive explanations.

Thank you very much for reviewing our paper. We have carefully read and addressed all the raised issues.

The authors may consider new analyses for (1) a multi-label classification task (i.e., PL, noPL, not recorded); and (2) retaining variables with missing values (that are not completely missing in the training set), or imputing these values prior to classification. These suggestions are explained below.

Thank you for the suggestions. Predicting a record/instance as being not recorded may not have any clinical value as it cannot be inferred whether the patient must be kept under observation or discharged as far as the aim of the study is concerned. It is also discussed further in the following responses from clinical viewpoint. It is also discussed why we chose to allow the learners (classifiers) to handle missingness rather than doing imputation as a separate step prior to classification. Briefly, our approach allows the learners to treat missing values as a piece of information in which missingness turns to an individual level that could contribute to splitting in tree-based models.

Data imputation, that is not embedded in the models as an alternative to our current approach, have serious limitations: 1) in model deployment, when a practitioner wants to use the model in clinic, they must replace the missing values with the practitioner’s best guess. This means that even if data imputation outside of the learner improves prediction performance in a study, model deployment of such remains obscure. 2) imputation may require looking at other features (variables) or values of the same feature across instances, and that could become a source of data leaks which could turn to overfitting or overestimation of the performance. 3) missingness by itself could convey useful information if there is a causality behind it, and it does not occur randomly.

As suggested, we have performed data imputation as an experiment to test whether there are any improvements by doing so. We used conditional multiple imputation from *mice* package in R. Our results did not demonstrate any improvements in predictions. This has been highlighted in the discussion section and detailed in the Supplementary information (see S7 Table).

Major comments
1. Why was plasma leakage not recorded in 172 patients? As reported in Table 1, there is a much higher proportion of missing outcome values in the non-dengue patients (26%) compared to the dengue patients (16%), which suggests the missing values are not missing at random and may indicate information of clinical importance. In this case, it might be more realistic to treat the prediction problem as a multi-class problem (i.e., PL, noPL, not recorded).

Regarding dengue patients, outcome for plasma leakage was not recorded when patients prematurely left the hospital against medical advice or withdrew consent for daily follow up. As for non-dengue patients seeing plasma leakage outside of dengue fever is very rare and as shown in a separate paper on the same dataset (DOI: 10.1371/journal.pone.0258388), their hospital stay is shorter than dengue patients. Since they feel better quickly and their clinical parameters improve, some of them would have been discharged sooner (before day 5 of fever) from hospital than plasma leakage can be confidently ruled out (e.g. by D7 after onset of fever). Noting that confirmation of dengue diagnosis is retrospective (by Rt-PCR), at the point of discharge the investigators did not know whether they had dengue or not. Therefore, for early discharges (which would mostly turn out to be non-dengue patients once RT-PCR is done), plasma leakage outcome was kept as not recorded, rather than absent. This explains why non-dengue patients had a higher missing value percentage for this outcome.

2. I’m unsure of the claim that removing instances with more than 50% of the features missing would reduce biased interpretation of feature contributions. Removing these instances would almost surely bias the interpretation because the missing values are presumably not missing at random. There are generally higher proportions of missing feature
values among the non-dengue patients compared to the dengue patients (Table 1), presumably because the tests are given more to the sicker patients. I expect that excluding these instances, rather than let the algorithms handle the missing data internally or impute thevalues, would limit the ability of the classifiers to learn the task.
Several papers show that imputing the missing values (e.g., with k-NN) can outperform the internal methods used by decision tree-based algorithms to treat missing data (e.g., [https://www.tandfonline](https://www.tandfonline/).com/doi/pdf/10.1080/08839514.2018.1448143). This paper showsthat adding missing-data perturbation prior to imputation can actually improve prediction accuracy in supervised classification tasks by regularizing the classifier.

The sentence about to reduce biased interpretation, refers to the possible bias in SHAP value computations. SHAP values are usually lower for features with more missing values than others and that could be a source of bias to rank features by SHAP values in presence of missingness. In other words, the features with fewer missing values could get higher ranks (importance) based on SHAP values solely because they have fewer missing values than others. We have This could be reduced by filtering out the samples (records) in which most of the features have missing values. The limitation about missingness is also discussed in references [22] and [23] which are added to the paper.

Regarding the missingness for non-dengue patients, there were two fields for viral load and serotype that were corrected to “-”. This and the previous response to the comment may remove the confusion regarding the missingness in the data. Data imputation may not necessarily improve prediction performance and conversely add bias to the models as explained in an earlier response. As you implicitly mentioned, missingness may by itself convey information especially in clinical setting and that suggests avoiding it or letting models such as random forest handle missing values as a unique level for splitting. Regarding the success of the imputation technique, it is noteworthy that the paper referred to here is about the effect of imputation of missing values for categorical features and that is more likely to succeed compared to the tasks in which most of the features are continuous variables as in our study.

3. Relatedly, in the discussion two contradictory statements are made:
that the proposed classifier can handle missing data and later, that many variables had be excluded due to missingness. I can see why variables that are completely missing in the training set (Dengue virus serotype and viral load) need to be dropped, but not understand
the intuition for dropping any other variables.

Thank you for the notion. Fig 1 clearly demonstrated the workflow to prepare the development set pertaining to missingness. From the blocks mentioning the missingness, it can be understood that not all the missingness are filtered out, and the workflow allows features and instances to include missing values in the development and test sets. The models are also stated in the paper to be able to handle missingness as embedded characteristics of their algorithms otherwise it would not work with instances including missing values. Therefore, we do not see any source of confusion or contradiction about missingness. The intuition to have a minimal set of selected features were 1) for practicality it is preferable to have less variables utilized and 2) our tests with greater number of features included did not lead to any significant improvement compared to the current results (S3 Table).

Minor comments
1. Pg. 4, typo: “focusses”

The word “focusses” is now changed to “focuses”.

2. Pg. 5: I don’t understand why the viral load and ultrasonography predictors are removed due to high costs. Is this because the non-dengue patients don’t get tested? Why are the ultrasound predictors not summarized in Table 1?

Dengue fever is clinically diagnosed and managed in Sri Lanka (in public hospitals such as the hospital from which patients were recruited for this study) and the diagnosis is often not confirmed by laboratory investigations due to high costs of tests. NS1 testing is only sporadically available at public hospitals and RT-PCR is almost never performed for clinical diagnosis. Diagnosis by RT-PCR and viral load estimations are only done for research purposes. This model of care is the most cost effective for a resource limited setting such as Sri Lanka as shown here in this published paper (DOI: 10.1371/journal.pone.0258388)

Similarly, ultrasonography is also a high cost investigation in this setting when considering the number of patients and the total number of scans needed per patient. This issue is explained in detail in this publication (doi: 10.1111/tmi.13594.)

On top of this since 2022, Sri Lankan economy has officially been declared bankrupt due to defaulting on sovereign debt obligations. This has further restricted funding for healthcare sector and the status quo is unlikely to improve in the next decade where the economic downturn is expected to continue.

However, we are not neglecting data such as ultrasonography findings and viral load. In fact, there are a lot of other data generated for this cohort such as full length viral genomes, host genome SNPs assessed by UK biobank array, differential cytokine expression in early infection, and pre-existing immunity to dengue or Zika (a structurally similar flavivirus that may behave as a dengue serotype) infection. We will use all of these in a second more comprehensive predictive model. However, while this future model may be of value in a scientific exploratory sense and have plausibly better performance, it has very limited use for being deployed to triage patients in a resource limited setting. In this paper and model we are strictly limiting ourselves to what can be done on the ground (at the cost of model performance).

3. Table 2: how is the variance of the performance metrics calculated?

The values for the performance measures indicate mean ± standard deviation of surrogate models from 2×5-fold cross validation in the outer loop. This information is now added to the footer of the Table 2.

4. Figure 3a.: Should the x-axis be labeled instead “1-Specificity” (the FPR)?

It is corrected, thank you for noticing.

Reviewer #2: The study aims to create a machine learning based model that can predict the occurrence of plasma leakage in patients with dengue fever based on accessible clinical and lab parameters. The study is well designed and of a huge clinical importance especially when thinking about implying machine learning models in order to decrease costs in developing countries.

Thank you very much for reviewing our paper. We have carefully read and addressed all the raised issues.

There are some points that needs to be clarified,
Regarding the training of the algorithms and the ensemble, how the algorithms parameters were tuned? How were the missing and unknown data handled?

For (hyper)parameter tuning, two bootstrapping repetitions of 100 sets selected based on random search over 80% of the instances in the development set to maximise the sensitivity. Regarding the missingness, we used the learner *randomForestSRC* as an implementation of random forest algorithm. From the documentation of the package, it is stated that “If a node splits on a variable with missing data, the variable's missing data is imputed by randomly drawing values from non-missing in-bag data. The purpose of this is to make it possible to assign cases to daughter nodes based on the split.” For LightGBM, missing values for continuous variables are assigned to the side of the splits that most reduces the loss. This description is added to the Supplementary information.

Some variables were excluded due to their high cost such as viral load and data from ultrasonography, I wonder if the authors tried to train the algorithms including these variables to see how their presence in the dataset would affect the created model performance.

Please see our response to reviewer 1 above, who had asked the same question.

Since there is no dataset for external validation, an internal-external validation approach could have been used [DOI: 10.1016/j.jclinepi.2015.04.005].

Our approach is based on nested cross validation in which we get an estimate of the prediction performance on a subset of the dataset for internal validation and the final model based on the development set is tested on a set-aside data (test set) for external validation.

The reference [18] is focusing on the random survival forests which is mainly used in survival analysis. I would recommend replacing it with a more-focusing reference on random forests in binary classification.

As suggested, the reference was replaced by a study on random forest with focus on classification.

Why there is a difference between the number of instances and number of patients? Does this mean that some patients had multiple instances?

Correct. Dengue is a dynamic illness and some tests such as full blood counts are done many times during admission for monitoring as the disease progresses.

Why the authors decided to go with an ensemble of both algorithms? Was the performance of the ensemble significantly better than the algorithms? I recommend comparing the performance of RF, LightGBM, and the ensemble to see if a model is statistically significantly better than the other [DOI: 10.1148/radiology.143.1.7063747]

The decision was upon our results that the ensemble design appears more robust than the single learners according to AUC and PRAUC. As suggested, we have provided this information to the benchmarking results (Table 2 and Fig 2). In addition, ensemble designs are usually preferred to individual learners to increase model robustness in cases where single algorithms fail to predict correctly. This does not mean that the ensemble design should necessarily increase the performance significantly but it rather provides a conservative approach for model robustness. Advantages of ensemble designs and stacking have been demonstrated in multiple studies such as <https://doi.org/10.1016/S0893-6080(05)80023-1>, <https://arxiv.org/abs/1204.0171>, and <https://arxiv.org/abs/2206.14477>.

The model performance on the test set shows that the AUC improved when selecting patients by Day of post fever onset. I think this is an important clinical point that needs to be discussed more in the discussion.

We added the following to the discussion: “Improved AUC was observed as day of post fever onset increased (Table 3). The observation may be explained by the increasing number of available patients by day (Fig 1) as well as an indication that the predictors approach to more certain areas in the feature space for the prediction.”

Since the aim of the study is creating a model that can help clinicians in developing countries, I think that the model should have been implemented into some kind of a practical tool such as webapp or a nomogram or a stratification flow chart so it can be used in practice.

As suggested, the model is made available as a simple webapp (see Fig S3 for a screenshot of the tool) and we added a direct link to download the model and its application programming interface in the data availability statement.

In the discussion section, the authors mentioned that simpler models such as naïve bayes, OneR, and logistic regression could not handle missingness and thus were not used in this study. Does this mean that their performance were comparable to the used algorithms [RF and LightGBM]?

No, it means that if we were planning to use those learners, we had to have complete records or do data imputation, that as we discussed, were not considered as a practical solution.

Reviewer #3: In the manuscript entitled "Development of a machine learning model for early prediction of plasma leakage in suspected dengue patients" by Marandi et al., the authors describe their work to develop a tool for plasma leakage prediction in the setting of dengue fever. This is a well performed study, with an adequate methodology. Unfortunatelly, the tool had not a high sensitivity in the prediction of plasma leakage; however, it showed to have a high specificity for that purpose. Therefore, although this is not a good tool to be taken into consideration alone in the decision of discharging patiens from hospitals, it may be useful to aid health care professionals to detect those patients who demand special vigilance due to the risk of clinical deterioration. Moreover, that tool does not require expensive diagnostic tests. These issues make the machine learning model developed by the authors a very useful tool specially in low-income countries affected by dengue virus, since the prediction of serious complications in patients infected with the virus is a challenging work for health
providers.

Thank you very much for reviewing our paper. We would also underline the pointed-out values of this work for clinical and research communities as well as directions for future investigations.

No further comments to address.
