# Supplementary material for: Development of a machine learning model for early prediction of plasma leakage in suspected dengue patients
Source: PLoS Negl Trop Dis. 2023 Mar 13;17(3):e0010758. doi: 10.1371/journal.pntd.0010758 (PMC10035900; doi:10.1371/journal.pntd.0010758)
Supplement: Attachment — Submitted filename: comments_r2.docx [file pntd.0010758.s014.docx]

Dear Editor,

Thank you for your feedback on the revised manuscript. We appreciate your support and the suggestions to further improve the quality of the paper.

Responses to comments:

The document highlights an interesting method and novel approach. Despite the favorable review, the manuscript still needs to tone down some of the conclusions and statements while acknowledging some of the limitations.

For instance, the missing data is an issue, yes it is a "real world" data, but analytically there are several approaches that could be used to address the missing data problem. Authors should indicate how, this would affect the results and their interpretations. Especially, plasma leakage was missing overall 20% but 25% for not dengue and 15% for dengue cases.

- Could you please comment on the effect of the differential missing for the outcome?

We have added a short description in the result section for the amount of missingness and how it differs between dengue and non-dengue patients. Regarding the differential missingness, we have added to the discussion that “The imputation supported the notion that the feature selection was not markedly influenced by the missingness in the data.”

- Authors indicated that "DENV5f-AS can handle missing data", could you please provide further details on the MICE implementation and how the S7 table compare to the main results in the main text?

For clarity, we have rephrased that statement to “On technical side, DENV_5f-AS_ has key strengths. As such, it can provide predictions for incomplete instances even with up to 50% missingness.” That has also been clarified where the AUC is compared between the cases of using MICE or not.

Also, overall results and implications from this ML exercise are not different from standard/traditional regression approaches with predictive approaches. This is, looking at hematological parameters and liver enzimes as predictors of severe dengue and plasma leakage has been the standard for the last several decades. My point, highlighting the effort of the authors, is to acknowledge that although you are contributing to the knowledge by illustrating the use of the novel methods, results are confirming some of the already stablished knowledge on the field. This should be addressed in the abstract, author summary, discussion and conclusion. Especially, because the results do not support that you provide "an artificial intelligence solution to predict plasma leakage in resource limited settings". The results showed that using an artificial intelligence tool, such ML, and with data from a resource limited setting, the authors observed a consistent prediction of plasma leakage by several known factors. These results are still the result from prediction, and in clinical settings, clinicians will only use the results form the literature but not necessarily recalculate these parameters using the data available to decide the course of treatment for a given patient under a given clinical presentation. To improve the quality of the literature we are getting out there for our audience, we must acknowledge and address our limitations accordingly.

We agree. The relevance of our observations is that the role of these predictors is further strengthened even after adjusting for missing data, non-linear associations and granularity (considering individual within-host and between-host data points without summarizing). This is the rephrased conclusion in the abstract: “*The early predictors of plasma leakage identified in this study are similar to those identified in several prior studies that used non-machine learning based methods. However, our observations strengthen the evidence base for these predictors by showing their relevance even when individual data points, missing data and non-linear associations were considered. Testing the model on different populations using these low-cost observations would identify further strengths and limitations of the presented model*.” The limitations of the ML approach are already discussed. We have also added the following statement to the 3^rd^ paragraph of discussion in keeping with the reviewers request “*While the predictors of plasma leakage reported here are mostly similar that reported in previous studies which did not use machine learning, our observations re-confirm the importance of these risk predictors even when individual within- and between-host observations (granularity), missing data and non-linear associations are considered. Thus our observations further strengthen the evidence base for some risk predictors of plasma leak*age”. We have also highlighted the fact that the model is limited to the scope of the variables under study and the population, and any generalization requires further studies.

Additional points:

- Please provide the % next to the missing data figures in table 1.

It is added as suggested.

- Please indicate how would you interpret the p-values for the False discovery rate correction for multiple testing in this context?

The description is added to the method section.

- Also, consider removing the p-values altogether or including them in the supplementary tables.

The p-values are removed as suggested.

- Authors summary starts with "We queried PubMed and Google Scholar for any publications between 1990 and 2022 using search terms such as “machine learning” or “artificial intelligence” or “prediction” and “plasma leakage”. which give the impression we are about to read s Systematic review, please consider rephrasing the section.

We have rephrased the summary to fix the raised issue.

Thank you again for your valuable input.
